# Association of frailty with influenza and hospitalization due to influenza among independent older adults: a longitudinal study of Japan Gerontological Evaluation Study (JAGES)

**DOI:** 10.1186/s12877-023-03979-y

**Published:** 2023-04-26

**Authors:** Kousuke Iwai-Saito, Koryu Sato, Jun Aida, Katsunori Kondo

**Affiliations:** 1 Division of International Health, Graduate School of Medical and Dental Sciences, Niigata University, 1-757 Asahimachi-Dori, Chuo-ku, Niigata City, 951-8510 Japan; 2 Department of Social Epidemiology, Graduate School of Medicine and School of Public Health, Kyoto University, Kyoto, Japan; 3 Department of Oral Health Promotion, Graduate School of Medical and Dental Sciences, Tokyo Medical and Dental University, 1-5-45 Yushima, Bunkyo City, Tokyo 113-8549 Japan; 4 Liaison Center for Innovative Dentistry, Tohoku University Graduate School of Dentistry, 4-1 Seiryo-Machi, Aoba Ward, 980-8574 Sendai City, Miyagi, Japan; 5 Department of Social Preventive Medical Sciences, Center for Preventive Medical Sciences, Chiba University, Chuo-Ku, Chiba, 260-8670 Japan; 6 Department of Gerontology and Evaluation Study, Center for Gerontology and Social Science, National Center for Geriatrics and Gerontology, Aichi, 7-430 Morioka-Cho, Obu, 474-8511 Japan

**Keywords:** Influenza, Hospitalization due to influenza, Frailty, And sex difference in infectious disease

## Abstract

**Background:**

It is unknown that whether frailty is a risk factor of influenza and the hospitalization among older adults, although it has been shown that frailty was associated with poor recovery from the hospitalization among those. We examined the association of frailty with influenza and the hospitalization and the effect by sex among independent older adults.

**Methods:**

We used the longitudinal data from the Japan Gerontological Evaluation Study (JAGES), performed in 2016 and 2019 and conducted in 28 municipalities in Japan. The target population comprised 77,103 persons aged ≥ 65 years who did not need assistance from the public long-term care insurance. Primary outcome measures were influenza and hospitalization due to influenza. Frailty was evaluated with the Kihon check list. We estimated the risk of influenza, the hospitalization, those risks by sex, and the interaction for frailty and sex using Poisson regression adjusting for covariates.

**Results:**

Frailty was associated with both influenza and the hospitalization among the older adults compared with nonfrail individuals after adjusting for covariates (influenza, frail: risk ratio {RR}: 1.36, 95% confidence interval {95% CI}: 1.20 − 1.53, and prefrail: RR: 1.16, 95% CI: 1.09 − 1.23; the hospitalization, frail: RR: 3.18, 95% CI: 1.84 − 5.57, and prefrail: RR: 2.13, 95% CI: 1.44 − 3.16). Male was associated with the hospitalization, but not associated with influenza compared to female (the hospitalization: RR: 1.70, 95% CI: 1.15 − 2.52 and influenza: RR: 1.01, 95% CI: 0.95 − 1.08). The interaction for frailty and sex was significant neither in influenza nor in the hospitalization.

**Conclusion:**

These results suggest that frailty is a risk of influenza and the hospitalization, that risks of the hospitalization are different by sex, but that the sex difference does not cause the effect heterogeneity of frailty on the susceptibility and severity among independent older adults.

**Supplementary Information:**

The online version contains supplementary material available at 10.1186/s12877-023-03979-y.

## Background

Influenza is a global public threat. The human influenza viruses usually cause moderate respiratory illness and infection of the upper respiratory tract. These patterns can result in pneumonia with progression to acute respiratory distress syndrome or in secondary bacterial infection and death because of respiratory failure. [1]. Congestive heart failure, coronary artery disease, cerebrovascular accidents, chronic obstructive pulmonary disease, asthma, diabetes mellitus, and end-stage renal disease are risk factors for influenza-related severe acute respiratory illness. [2]. It has been estimated that ~ 389,000 respiratory deaths were associated with influenza worldwide each year during the study period, ~ 2% of all annual respiratory deaths, and 67% of these were among older adults aged ≥ 65 years. [3].

Frailty is defined as a state of increased vulnerability to stressors, which develops as a consequence of an age-related decline in multiple physiological and psychological systems, including the central nervous system, endocrine system, skeletal muscle, and immune system. [4, 5]. Recent reports have shown that the pooled prevalence rates of frail older adults in 62 countries were 11%–13% and 22%–26% for physical frailty measures and the deficit accumulation model, respectively. [6].

Frailty is associated with susceptibilities and severities of infectious diseases. Lengelé et al. showed that frail was associated with higher incidences of COVID-19 compared to nonfrail among community-dwelling older adults over 65 years. [7] Hewitt et al. reported that the clinical frailty score predicted in-hospital mortality among older adults with COVID-19 better than age or comorbidity. [8]. We recently reported that frailty was associated with susceptibility to pneumonia and the hospitalization among independent older adults aged ≥ 65 years. [9]. Luo et al. showed that that frailty was associated with severity and mortality of older adults aged ≥ 65 years hospitalized due to pneumonia. [10]. Rodriguez et al. showed that prefrailty was associated with HIV-associated mild neurocognitive disorder in older adults. [11].

Lees et al. recently reported that frailty increased poor recovery in the older adults who are hospitalized because of influenza-related illnesses. [12]. However, it is unknown that frailty is a risk of influenza and the hospitalization among independent older adults. Therefore, this study examined whether frailty was associated with influenza and the hospitalization among independent older adults aged ≥ 65 years. Furthermore, several reports showed that both influenza- and frailty-associated mortalities are different among sex. [13–15]. This study also hypothesized that sex difference has an effect on influenza and the hospitalization and the relationship between influenza and frailty.

## Methods

### Study sample and design

This study had a longitudinal design and used data from the Japan Gerontological Evaluation Study (JAGES). JAGES aims to survey the social determinants of health among non-institutionalized and functionally independent persons aged 65 and older who did not receive public long-term care insurance (LTCI) benefits. This public program is described as follows: “The LTCI system was introduced in Japan in 2000 to address the demands of older persons with disabilities based on the concept of a user-oriented social insurance system with support for independence. Older people with a certification for LTCI service needs can utilize facility services, in-home services, and community-based services depending on their physical and cognitive impairments.” [16]. This longitudinal study used the panel data from two surveys. Survey subjects were selected in units of cities, towns, and villages. All individuals were generally included if the number was less than 5000, except in the case of budgetary restrictions or the size of the city, town, or village. In the case that this included more than 5000 individuals, questionnaire booklets were distributed to 5000 randomly-selected individuals. [17].

### Analytical sample

**Fig. 1:**
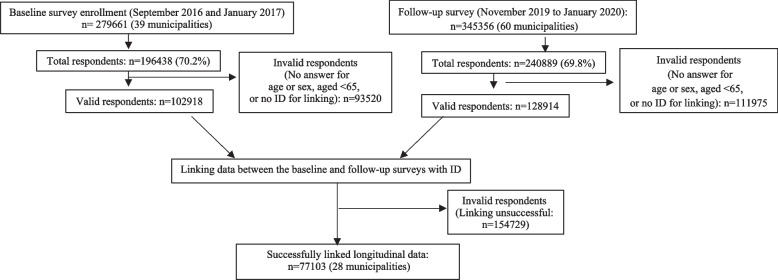
Flowchart of participants and the exclusion criterion

The baseline survey was conducted between September 2016 and January 2017 mailed to community-dwelling individuals in 39 municipalities and the data was obtained from self-reported questionnaires among 196,438 of 279,661 individuals (70.2%) responded to the survey (Fig. 1). After excluding data from older adults who had been certified as needed the long- term care at the survey, those who aged < 65 years, no ID number, and those who did not answer any question at all, the number of participants in the “JAGES2016” data attributed to 102,918. [18]. The follow-up survey was conducted between November 2019 to January 2020. Self-administered questionnaires used for the follow-up survey were subsequently mailed to a total of 345,356 community-dwelling individuals in 60 municipalities and the data was obtained from self-reported questionnaires among 240,889 individuals (69.8%) responded to our mail. [19] After excluding data from older adults who had been certified as needed the long- term care at the survey, those who aged < 65 years, no ID number, and those who did not answer any question at all, the number of participants in the “JAGES2019” data attributed to 128,914. After excluding data from older adults who we could not merge individual data between in the baseline and follow-up surveys tab with the identification number, the number of participants in the “JAGES 2016–2019” panel data attributed to 77,103.

### Influenza and hospitalization due to influenza

Influenza and hospitalization due to influenza in the past year were assessed with two questions in the follow-up survey. The first asked the participants, “Did you get sick in the past year?” and provided the following possibilities: “Influenza,” “Pneumonia,” and “neither.” The second question asked, “If you were sick with influenza or pneumonia, were you hospitalized due to it?” and instructed them to select responses from the following options: “Not hospitalized,” “Hospitalized due to influenza,” “Hospitalized due to pneumonia,” “Contracted influenza while I was hospitalized for another disease,” and “Developed pneumonia while I was hospitalized for another disease.” The participants who answered “Influenza” to the first question and “Hospitalized due to influenza” or “Hospitalized due to pneumonia” to the second one were considered as hospitalized due to influenza because influenza can cause either primary viral or secondary bacterial pneumonia. [20].

### Frailty and sex

Frailty was assessed using the Kihon Checklist (KCL), which was developed by the Japanese Ministry of Health, Labor and Welfare to identify older adults who were at risk for requiring LTCI in the baseline survey. [21]. KCL was included in the self-administered questionnaires of the JAGES 2016 wave. KCL consists of 25 questions grouped into seven categories: instrumental activities of daily living, physical strength, nutritional status, oral function, homeboundness, cognitive function, and depressive mood. [22]. The scores of the KCL correlated with validated assessments of physical strength, nutritional state, cognitive function, depressive mood, and the number of frailty phenotypes defined by the Cardiovascular Health Study (CHS) criteria. [22]. Frailty was categorized into three groups based on the total score of KCL: the total score 0–3 was categorized as nonfrail, 4–7 as prefrail, and ≥ 8 as frail. The categorization was validated with the prefrail and frail categories established by the CHS criteria. [22]. Sex was assessed by asking participants “The following questions are about your personal characteristics. Circle the number of the answer the best applies.” And they were introduced to choose one of two answers as follows: male and female.

### Covariates

Age was classified into two groups (65–74 years and ≥ 75 years). Educational attainment was categorized into five groups: < 6 years, 6–9 years, 10–12 years, ≥ 13 years, and others. Equivalized income was calculated by dividing the normalized household gross income in 2015 by the square root of the number of household members, and then, it was categorized into five groups: < 0.5, 0.50–0.99, 1.00–1.99, 2.00–3.99, and ≥ 4.00 million yen. Household structure was assessed by asking respondents “Who do you live with?” with the following options: “no one,” “spouse,” “son,” “daughter,” “spouse of child,” “grandchild,” “brother or sister, “father,” “mother,” “father-in-law,” “mother-in-law,” and “other.” Responses were classified into six groups: living alone, living with a spouse, living with children, living with a spouse and children, living in a three-generation household (living with at least one of the sons/daughters/son’s or daughter’s spouse, and grandchildren), and living in a household structure other than the above five categories. [18]. Smoking status was assessed with the question “Do you smoke cigarettes?” and the participants selected appropriate answers from among the following items: “Never smoked,” “Quit smoking ≥ 5 years ago,” “Quit smoking < 5 years ago,” “Smoke sometimes,” and “Smoke almost every day.” Chronic diseases including stroke, heart disease, diabetes, respiratory disease, kidney or prostate disease, or blood or immune disease are associated with the severity of influenza. [20, 23]. The chronic diseases were assessed by asking “Circle the number of all diseases for which you are currently receiving treatment or experiencing after-effects.” and the responders were scored “1” if they circled at least one of the eight diseases, otherwise they scored “0”. The influenza vaccination program was initiated in 2001 to prevent the severe illness among older adults who aged ≥ 65 in Japan. [24]. The nation-wide proportion of the vaccination was 65.6% in the 2019–2020 season. [25] The vaccination is encouraged by municipalities, but is not legally binding. Most of the municipalities provide subsidies for the vaccination, but the amount varies among those. [24] Influenza vaccination was assessed by asking participants “Have you had flu shot in the past year?” Civic participation, reciprocity, and number of friends met in the past month were used to assess social relationships, which had been potentially associated with opportunities for influenza virus infection and the associations had been different among sex. [26, 27]. Civic participation was assessed by asking how often they took part in sports groups or clubs, volunteer groups, hobby activity groups, study or cultural groups, and activities to teach skills or pass on experiences to others (frequencies were divided as follows: ≥ 4 times per week, 2–3 times per week, once a week, 1–3 times per month, several times per year, or never). Civic participation was scored “1” if respondents participated in any of the five social groups once a month or more often and scored “0” if respondents participated in these social groups less than once a month. Reciprocity was assessed with questions related to emotional or instrumental social support: “Do you have someone who listens to your concerns and complaints?” “Do you listen to someone’s concerns and complaints?” and “Do you have someone who looks after you when you are sick for a few days?” Reciprocity was scored “1” if respondents answered “one or more people” to any of the questions and scored “0” if respondents answered “no one” to all of the three questions. The number of friends met in the past month was assessed by asking “How many friends or acquaintances have you met in the past month? Count the same person as one no matter how many times you meet.” The number of friends met in the past month was categorized into five groups: 0, 1–2, 3–5, 6–9, and ≥ 10 friends. The dummy variable was generated for the 28 municipalities with the arbitrary assigned numbers.

### Statistical analyses

A directed acyclic graph (DAG) was created to identify a minimal sufficient adjustment set (MSAS) of potential confounders to estimate a direct effect of frailty on influenza and hospitalization due to influenza among older adults. An online tool, DAGitty v3.0 (http://www.dagitty.net), was used to create the DAG. The DAG analyses revealed that the MSASs to estimate the effects were age, sex, marital status, educational attainment, equivalized income, household structure, smoking status, high-risk disease, influenza vaccination, civic participation, reciprocity, number of friends met in the last month, and the municipalities. [28]. For missing response (Supplemental Table 1), we presumed that the missing pattern of the original data set was missing at random. Twenty imputed data sets were generated for handling missing data by the Markov chains. [29]. The poisson regression model was used to estimate risk ratio (RR) and 95% confidence interval (CI) for the association between influenza or the hospitalization and frailty based on robust standard errors after adjusting for age, sex, marital status, educational attainment, equivalized income, household structure, smoking status, high-risk disease, influenza vaccination, civic participation, reciprocity, number of friends met in the last month, and the municipalities. The association between influenza or the hospitalization and sex was estimated after adjusting for the frailty, age, marital status, educational attainment, equivalized income, household structure, smoking status, high-risk disease, influenza vaccination, civic participation, reciprocity, number of friends met in the last month, and the municipalities. The interaction was estimated in the same regression model including the interaction term for frailty and sex. Sensitivity analyses were conducted using the complete data without missing data and the estimations were calculated in the same models as those using the imputed data sets. All P values were two-tailed, and the significance was set at 5%. Stata version 17.1 (Lightstone Corp., College Station, TX, USA) was used for all analysis.

### Patient and public involvement

No patients were involved in the development of the research question, study design, or data interpretation in this study.

## Results

Table 1 shows baseline characteristics of independent older adults aged ≥ 65 years with or without influenza or hospitalization due to influenza at the follow-up.

**Table 1:** Baseline characteristics of independent older adults aged ≥ 65 years with or without influenza or hospitalization due to influenza at the follow-up survey

		Influenza (*n* = 76,731) ^a^				Hospitalization due to influenza (*n* = 76,734) ^a^			
		No (*n* = 72,096, 94.0%)		Yes (*n* = 4635, 6.0%)		No (*n* = 76,560, 99.8%)		Yes (*n* = 174, 0.2%)	
		n	%	n	%	n	%	n	%
Frailty	Nonfrail	36,913	51.2	2220	47.9	39,046	51.0	50	29.0
	Prefrail	31,001	43.0	2104	45.4	32,997	43.1	100	57.4
	Frail	4182	5.8	311	6.7	4517	5.9	24	13.6
Age group	65‒74	44,339	61.5	3022	65.2	47,314	61.8	61	35.1
	≥ 75	27,757	38.5	1613	34.8	29,246	38.2	113	64.9
Sex	Male	34,318	47.6	2188	47.2	36,366	47.5	108	62.1
	Female	37,778	52.4	2447	52.8	40,194	52.5	66	37.9
Educational attainment (years)	< 6	433	0.6	19	0.4	459	0.6	3	1.8
	6–9	19,250	26.7	1154	24.9	20,365	26.6	56	32.0
	10–12	30,929	42.9	1975	42.6	32,844	42.9	69	39.9
	≥ 13	21,052	29.2	1465	31.6	22,509	29.4	43	24.9
	Others	360	0.5	23	0.5	383	0.5	2	1.3
Equivalized income, million yen	< 0.5	5984	8.3	357	7.7	6278	8.2	18	10.3
	0.50–0.99	20,908	29.0	1298	28.0	22,126	28.9	55	31.6
	1.00–1.99	14,996	20.8	1052	22.7	16,001	20.9	30	17.2
	2.00–3.99	9949	13.8	663	14.3	10,565	13.8	17	9.8
	≥ 4.00	20,259	28.1	1265	27.3	21,513	28.1	54	31.0
Marital status	Married	54,505	75.6	3652	78.8	58,032	75.8	134	77.0
	Widowed	12,328	17.1	663	14.3	12,939	16.9	26	14.9
	Divorced	3100	4.3	199	4.3	3292	4.3	9	5.2
	Never married	2091	2.9	116	2.5	2220	2.9	5	2.9
	Other	72	0.1	5	0.1	77	0.1	-	-
Household structure (Living with who or by alone)	Spouse	33,597	46.6	2211	47.7	35,677	46.6	79	45.4
	Alone	6777	9.4	357	7.7	7120	9.3	17	9.8
	Offspring	4758	6.6	246	5.3	4976	6.5	9	5.2
	Spouse and offspring	10,814	15.0	723	15.6	11,484	15.0	22	12.6
	Three-generation household	7065	9.8	514	11.1	7579	9.9	27	15.5
	The other households other than above	9084	12.6	584	12.6	9647	12.6	20	11.5
Smoking status	Smoke almost everyday	6272	8.7	375	8.1	6584	8.6	19	10.9
	Smoke sometimes	1009	1.4	70	1.5	1072	1.4	5	2.9
	Quit smoking < 5 years ago	2379	3.3	134	2.9	2526	3.3	10	6.0
	Quit smoking ≥ 5 years ago	19,538	27.1	1298	28.0	20,824	27.2	53	30.4
	Never smoked	42,897	59.5	2753	59.4	45,553	59.5	87	49.8
High-risk disease	No	53,207	73.8	3370	72.7	56,425	73.7	108	61.8
	1 or more	18,889	26.2	1265	27.3	20,135	26.3	66	38.2
Influenza vaccination	No	29,559	41.0	1664	35.9	31,236	40.8	49	28.4
	Yes	42,537	59.0	2971	64.1	45,324	59.2	125	71.6
Civic participation	No participation	41,095	57.0	2443	52.7	43,410	56.7	108	62.2
	1 or more	31,001	43.0	2192	47.3	33,150	43.3	66	37.8
Reciprocity	No reciprocity	2019	2.8	102	2.2	2067	2.7	8	4.7
	1 or more	70,077	97.2	4533	97.8	74,493	97.3	166	95.3
Number of friends met in the last month	0	5407	7.5	287	6.2	5665	7.4	17	9.5
	1–2	12,184	16.9	714	15.4	12,862	16.8	39	22.3
	3–5	17,375	24.1	1089	23.5	18,451	24.1	45	25.9
	6–9	10,310	14.3	667	14.4	11,025	14.4	21	11.9
	10 or more	26,748	37.1	1877	40.5	28,557	37.3	53	30.3

^a^numbers of participants with or without influenza or the hospitalization were not equal to the number of total participants (77,103) because the numbers of influenza or the hospitalization varied among the imputations and those were adjusted to the minimum numbers

Frail or prefrail older adults in the baseline were more likely to contract influenza in the follow-up than nonfrail those. Older adults with the following characteristics in the baseline were more likely to contract influenza than their counterparts: aged 65–74 years, being female, had ≥ 13 years education, had 1.00–1.99 or 2.00–3.99 million equivalized income per year, being married, lived with a spouse, lived with a spouse and offspring, or lived in a three-generation household, smoked sometimes or quit smoking ≥ 5 years ago, had ≥ 1 high-risk diseases, received an influenza vaccination, had ≥ 1 civic participation, had ≥ 1 reciprocity, or met ≥ 6 friends in the last month.

A greater percentage of frail or prefrail older adults were hospitalized due to influenza than nonfrail ones. Older adults with the following characteristics were more likely to be hospitalized due to influenza than their counterparts: aged ≥ 75 years, being male, had < 6 years, 6–9 years, or other education, had < 0.5, 0.50–0.99, or ≥ 4.00 million income, being married or divorced, lived alone or lived in a three-generation household, smoked almost every day, sometimes, quit smoking < 5, or ≥ 5 years ago, had ≥ 1 high-risk diseases, received influenza vaccination, had no civic participation/no reciprocity, or met 0, 1–2, or 3–5 friends in the last month.

Table 2 shows the RRs and 95% CIs of the association between influenza or hospitalization due to influenza and frailty or sex among older adults.

**Table 2:** Associations of influenza or the hospitalization with frailty or sex among older adults aged ≥ 65 years (*n* = 77,103)

	Influenza				Hospitalization due to influenza			
	Unadjusted		Adjusted		Unadjusted		Adjusted	
	RR	95% CI	RR	95% CI	RR	95% CI	RR	95% CI
Nonfrail	1.00	Reference	1.00	Reference	1.00	Reference	1.00	Reference
Prefrail	1.12	1.06 − 1.19	1.16	1.09 − 1.23	2.36	1.60 − 3.47	2.13	1.44 − 3.16
Frail	1.23	1.10 − 1.38	1.36	1.20 − 1.53	4.21	2.53 − 6.99	3.18	1.84 − 5.57
Female	1.00	Reference	1.00	Reference	1.00	Reference	1.00	Reference
Male	0.99	0.93 − 1.04	1.01	0.95 − 1.08	1.84	1.36 − 2.49	1.70	1.15 − 2.52

*RR* risk ratio, *95% CI* 95% confidence interval. For the estimation of the associations between influenza or the hospitalization and frailty, RRs and 95% CIs were adjusted for age, sex, marital status, educational attainment, equivalized income, household structure, smoking status, high-risk disease, influenza vaccination, civic participation, reciprocity, numbers of friends met in the last month, and the municipalities. For the estimation of the associations between influenza or the hospitalization and sex, RRs and 95% CIs were adjusted for frailty, age, marital status, educational attainment, equivalized income, household structure, smoking status, high-risk disease, influenza vaccination, civic participation, reciprocity, numbers of friends met in the last month, and the municipalities

After adjusting for all covariates, frail or prefrail was associated with influenza compared with nonfrail (frail: RR: 1.36, 95% CI: 1.20 − 1.53 and prefrail: RR: 1.16, 95% CI: 1.09 − 1.23). Frail or prefrail was associated with the hospitalization (frail: RR 3.18, 95% CI 1.84‒5.57 and prefrail: RR: 2.13, 95% CI 1.44–3.16). Male was associated with the hospitalization, but not with influenza compared to female after adjusting for frailty and the other covariates, (the hospitalization: RR: 1.70, 95% CI 1.15 − 2.52 and influenza: RR 1.01, 95% CI 0.95 − 1.08). The results of the sensitivity analysis conducted on the complete data were nearly the same as the results in the mutated data sets except for the RR of the hospitalization was higher in prefrail than frail. The association was significant neither in influenza nor in the hospitalization with male compared to female after adjusting for the covariates in the complete data. (Supplemental Table 2).

Table 3 shows RRs and 95% CIs of interactions for frailty and sex in influenza or the hospitalization.

**Table 3:** Interactions for frailty and sex in influenza or in the hospitalization (*n* = 77,103)

	Influenza				Hospitalization due to influenza			
	Unadjusted		Adjusted		Unadjusted		Adjusted	
	RR	95% CI	RR	95% CI	RR	95% CI	RR	95% CI
Nonfrail female	1.00	Reference	1.00	Reference	1.00	Reference	1.00	Reference
Prefrail male	1.07	0.95 − 1.21	1.08	0.96 − 1.22	0.93	0.42 − 2.03	0.94	0.43 − 2.05
Frail male	1.14	0.90 − 1.43	1.13	0.90 − 1.43	1.08	0.37 − 3.14	1.05	0.36 − 3.04

*RR* risk ratio, *95% CI* 95% confidence interval. RRs and 95% CIs of the interactions were adjusted for sex, frailty, age, marital status, educational attainment, equivalized income, household structure, smoking status, high-risk disease, influenza vaccination, civic participation, reciprocity, and numbers of friends met in the last month, and the municipalities

The interactions for frailty and sex were neither significant in influenza nor the hospitalization (influenza: frail male: RR: 1.13, 95% CI: 0.90 − 1.43; prefrail male: RR: 1.08: 0.96 − 1.22 and the hospitalization: frail male: RR: 1.05, 95% CI: 0.36 − 3.04; prefrail male: RR: 0.94, 95% CI 0.43 − 2.05). The interactions for frailty and sex were neither significant in influenza nor the hospitalization as same with those in the imputed data sets (Supplemental Table 3).

## Discussion

This longitudinal study analyzed whether frailty was associated with influenza and hospitalization due to influenza among independent older adults aged ≥ 65 years. After adjusting for all covariates, this analysis showed that both frail and prefrail were associated with both influenza and the hospitalization. Male was associated with the hospitalization, but not with influenza compared to female. The interactions for frailty and sex were neither significant in influenza nor in the hospitalization. These results suggest that frailty is a risk of influenza and the hospitalization, that the risks of hospitalization are different by sex, but that the sex difference does not cause the effect heterogeneity between influenza and frailty among independent older adults.

Lees et al. recently showed that frailty predicts poor recovery of older adults who were hospitalized due to influenza illness in the prospective cohort study. [12]. Our result showed that frailty was associated with influenza and the hospitalization among independent older adults. These results suggest that frailty is not only a risk of poor recovery from the hospitalization in older adults, but also a risk of influenza and the hospitalization in independent older adults. These results indicate that frailty should be considered as a risk factor of influenza which potentially increases the susceptibility and severity same as high-risk diseases. [2].

Several reports suggest a causal role from frailty to onset of infectious diseases and to their severity by showing that frailty is associated with susceptibility to and severity of infectious diseases in longitudinal studies. [7, 8, 10, 12, 30]. Our result supports that the direction of causal role. Given that frail is defined as a state of increased vulnerability to stress with consequence of an age-related declines of physiological and psychological systems, [4, 5] it is reasonable that frail older adults are more susceptible to infectious diseases and at higher risk of those severe illnesses than that nonfrail those. On the other hand, several reports showed that hospitalization due to pneumonia was associated with frailty including declined instrumental or basic activity of daily living, cognitive impairment, or depression in older adults, suggesting that the opposite direction of the causal relationship. [31–34]. There may be the both directions of the causal relationship between infectious diseases and frailty, and infectious diseases and frailty may be risk factors for each other.

There were sex differences in immunity against virus infections, and it affects susceptibilities to and severities of viral infectious diseases. [13]. Human ovaries and testes secrete differential concentrations of sex steroids, including estrogens, progesterone, and androgens. Immune cells including myeloid cells and lymphocytes which express receptors for sex steroids, which can transcriptionally regulate gene expression and responses of immune cells following viral infection. Consequently, there are differences in activation of the immune system against infections by sexes. [13]. Wang et al. showed that male were associated with the severe illness of influenza than female among older adults aged ≥ 65 during typical seasonal influenza epidemics. [35] Our results showed that male was associated with the hospitalization, but not with influenza compared to female after adjusting the covariates including frailty among independent older adults. These results suggest that the sex difference in activation of immune system may have different effects on severity of influenza, but not on the susceptibility among older adults. These results indicate that male should be considered as a risk factor which can increase the severity of influenza among older adults. On the other hand, our result showed that the interaction for frailty and sex was neither significant in influenza nor in the hospitalization. It is suggested that sex difference causes different severities of influenza and that frail older men have higher mortality compared to those women. [13–15]. However, given potential confounders for the interaction, sex differences may not cause a heterogeneity in the effect of frailty on influenza susceptibility and severity.

Our study had limitations. First, the nature of the observational study did not clarify causality because of unmeasured confounders. However, this study attempted to adjust for major confounding variables from the individuals and influenza. Second, influenza infection and the hospitalization were assessed by self-reports. Therefore, we have no information that how influenza was diagnosed and the hospitalization was decided by a physician. However, it is unlikely that they arbitrarily diagnosed themselves with influenza or non-medical professionals did so and they trusted it because most Japanese citizens have good medical access due to the national health coverage and rapid diagnostic tests with high sensitivity and specificity are used to detect influenza antigens by medical professionals in all cases of suspected influenza. [36, 37]. Third, these findings cannot be generalized to people who had been certified as needing long-term care. Forth, it is possible that older adults were automatically excluded from the survey if their influenza was too severe to answer the questionnaire. Fifth, occurrences of seasonal influenza and the hospitalization in the 2017–2018 season between the baseline and follow-up surveys were not observed in this study and it could cause underestimation or overestimation of the relationship between influenza and frailty. Sixth, an informational bias may have appeared in the survey for smoking status because of self-reported-based assessments. [38]. This bias may have obscured the relationship between smoking status and influenza susceptibility or severity. Seventh, recall bias may occur in the survey regarding having received the influenza vaccination in the previous year (Table 1). This bias may have affected the relationship between vaccination status and influenza susceptibility or severity.

## Conclusion

It was unknown that whether frailty was a risk of influenza susceptibility and severity among independent older adults. This study examined whether frailty was associated with influenza and the hospitalization in functionally independent older adults aged ≥ 65 years and whether sex difference have an effect on those incidents. Our results showed that both frailty and prefrailty were associated with influenza and the hospitalization after adjusting for all covariates, that male was associated with the hospitalization, but not with influenza compared to female, but that the interaction for frailty and sex was neither significant in influenza nor in the hospitalization. Our results suggest that frailty is a risk to increase susceptibility to influenza and its severity, that risk of the hospitalization is different by sex, but that sex does not cause a heterogeneity in the effects of frailty on the susceptibility and severity. Health care policies to reduce influenza mortality and health care providers for older adults should recognize that frailty not only interferes with tertiary prevention to recover from hospitalization for the severe illness, but also with the primary one to prevent the onset of influenza and the secondary one to prevent the severe illness among older adults.

## Supplementary Information

**Additional file 1:**

## Data Availability

Data are available upon sensible request. The datasets of JAGES, which were used in this research, are available from the corresponding author upon reasonable request. All inquiries should be addressed to the data management committee via e-mail: dataadmin.ml@jages.net. All JAGES datasets have ethical or legal restrictions for public deposition because of the inclusion of sensitive information from the human participants. Following the regulations of local governments, which cooperated in the survey, the JAGES data management committee has imposed restrictions upon the data.
