# Supplementary material for: Association of frailty with influenza and hospitalization due to influenza among independent older adults: a longitudinal study of Japan Gerontological Evaluation Study (JAGES)
Source: BMC Geriatr. 2023 Apr 26;23:249. doi: 10.1186/s12877-023-03979-y (PMC10131426; doi:10.1186/s12877-023-03979-y)
Supplement: Supplementary file 1 — Additional file 1: [file 12877_2023_3979_MOESM1_ESM.docx]

Supplemental Table 1 Baseline characteristics of the older adults with influenza or the hospitalization with missing (n = 77103)

|  |  | Influenza (%) | | | Hospitalization due to influenza (%) | | |
| --- | --- | --- | --- | --- | --- | --- | --- |
|  |  | No  (n=60499) | Yes  (n=4652) | Missing  (n=11952) | No  (n=64666) | Yes  (n=176) | Missing  (n=12,261) |
| Frailty | Nonfrail | 43.9 | 40.4 | 30.3 | 43.6 | 18.2 | 30.4 |
|  | Prefrail | 39.1 | 42.3 | 39.1 | 39.2 | 48.9 | 39.0 |
|  | Frail | 5.2 | 6.6 | 7.6 | 5.3 | 13.1 | 7.7 |
|  | Missing | 11.7 | 12.7 | 22.9 | 11.7 | 19.9 | 22.9 |
| Age group | 65‒74 | 64.3 | 66.5 | 47.1 | 64.3 | 35.2 | 47.5 |
|  | ≥75 | 35.7 | 35.5 | 52.9 | 35.4 | 64.8 | 52.5 |
| Sex | Male | 49.4 | 47.2 | 38.1 | 49.2 | 62.5 | 38.1 |
|  | Female | 50.5 | 52.8 | 61.9 | 50.6 | 37.5 | 61.9 |
|  | Missing | 0.01 | 0.0002 | 0.01 | 0.01 | No missing | 0.008 |
| Educational attainment  (years) | <6 | 0.4 | 0.3 | 1.0 | 0.4 | 1.7 | 1.0 |
|  | 6–9 | 24.2 | 25.3 | 38.0 | 24.1 | 31.3 | 38.0 |
|  | 10–12 | 43.5 | 43.2 | 38.5 | 43.4 | 39.2 | 38.4 |
|  | ≥13 | 30.8 | 32.1 | 20.5 | 30.8 | 25.0 | 20.6 |
|  | Others | 0.5 | 0.5 | 0.8 | 0.5 | 1.1 | 0.8 |
|  | Missing | 0.5 | 0.5 | 1.2 | 0.5 | 1.7 | 1.2 |
| Equivalized income, million yen | <0.5 | 7.5 | 7.9 | 12.7 | 7.5 | 10.2 | 12.6 |
|  | 0.50–0.99 | 29.3 | 28.5 | 27.6 | 29.1 | 31.8 | 27.7 |
|  | 1.00–1.99 | 21.8 | 23.1 | 15.7 | 21.8 | 17.0 | 15.7 |
|  | 2.00–3.99 | 14.8 | 14.6 | 8.7 | 14.7 | 10.2 | 8.8 |
|  | ≥4.00 | 26.7 | 27.9 | 35.3 | 26.7 | 30.7 | 35.3 |
| Marital status | Married | 76.0 | 79.4 | 67.0 | 76.0 | 73.9 | 67.2 |
|  | Widowed | 15.7 | 14.4 | 22.7 | 15.5 | 14.8 | 22.5 |
|  | Divorced | 4.2 | 4.3 | 4.6 | 4.2 | 5.1 | 4.6 |
|  | Never married | 2.9 | 2.5 | 2.5 | 2.9 | 2.8 | 2.5 |
|  | Other | 0.5 | 0.5 | 1.0 | 0.5 | 1.1 | 1.0 |
|  | Missing | 0.7 | 0.8 | 2.2 | 0.7 | 2.3 | 2.2 |
| Household structure  (Living with who or by alone) | Spouse | 47.4 | 48.5 | 41.6 | 47.3 | 45.5 | 41.8 |
|  | Alone | 9.3 | 8.0 | 10.6 | 9.2 | 10.2 | 10.6 |
|  | Offspring | 6.2 | 5.4 | 8.4 | 6.2 | 5.1 | 8.2 |
|  | Spouse and offspring | 15.4 | 15.8 | 12.4 | 15.4 | 12.5 | 12.4 |
|  | Three-generation household | 9.6 | 11.3 | 11.2 | 9.6 | 15.3 | 11.3 |
|  | Other Households structures | 12.1 | 12.9 | 15.8 | 12.1 | 11.4 | 15.7 |
| Smoking status | Smoke almost everyday | 8.9 | 8.2 | 6.9 | 8.9 | 10.2 | 6.9 |
|  | Smoke sometimes | 1.4 | 1.5 | 1.5 | 1.4 | 2.8 | 1.5 |
|  | Quit smoking <5 years ago | 3.1 | 2.8 | 2.7 | 3.1 | 5.1 | 2.7 |
|  | Quit smoking ≥5 years ago | 27.4 | 27.9 | 21.5 | 27.4 | 28.4 | 21.5 |
|  | Never smoked | 57.9 | 59.9 | 64.4 | 57.8 | 49.4 | 64.4 |
|  | Missing | 1.2 | 1.6 | 3.0 | 1.2 | 4.0 | 3.0 |
| High-risk disease | No | 70.4 | 70.3 | 66.7 | 70.1 | 58.5 | 66.7 |
|  | 1 or more | 25.8 | 27.7 | 27.4 | 25.8 | 38.1 | 27.4 |
|  | Missing | 3.8 | 4.0 | 5.9 | 3.8 | 3.4 | 5.9 |
| Influenza vaccination | No | 39.6 | 34.2 | 30.5 | 39.1 | 27.3 | 30.5 |
|  | Yes | 57.0 | 64.5 | 63.5 | 57.2 | 70.5 | 63.6 |
|  | Missing | 3.4 | 3.2 | 6.0 | 3.4 | 2.3 | 6.0 |
| Civic participation | No participation | 38.9 | 36.0 | 33.3 | 38.6 | 36.9 | 33.4 |
|  | 1 or more | 40.7 | 43.7 | 29.8 | 40.8 | 34.1 | 30.1 |
|  | Missing | 20.4 | 22.3 | 36.9 | 20.4 | 29.0 | 36.6 |
| Reciprocity | No reciprocity | 1.0 | 0.8 | 1.1 | 1.0 | 2.3 | 1.1 |
|  | 1 or more | 96.2 | 98.3 | 92.8 | 96.0 | 93.2 | 92.9 |
|  | Missing | 2.8 | 2.9 | 0.0 | 2.8 | 4.5 | 6.0 |
| Number of friends met in the last month | 0 | 7.3 | 6.0 | 6.6 | 7.2 | 9.1 | 6.6 |
|  | 1–2 | 16.3 | 15.4 | 17.0 | 16.2 | 22.2 | 16.9 |
|  | 3–5 | 23.1 | 23.3 | 25.6 | 23.0 | 24.4 | 25.7 |
|  | 6–9 | 13.9 | 14.3 | 14.1 | 13.8 | 11.9 | 14.2 |
|  | 10 or more | 37.7 | 40.9 | 32.5 | 37.8 | 29.5 | 32.4 |
|  | Missing | 1.7 | 2.1 | 4.2 | 1.7 | 2.8 | 4.2 |

Numbers represent percentages for each group.

-: no missing

Supplemental Table 2 Associations between influenza or the hospitalization and frailty among the older adults in the complete data

|  | Influenza (n=43348) | | | | Hospitalization due to influenza (n=43178) | | | |
| --- | --- | --- | --- | --- | --- | --- | --- | --- |
|  | Unadjusted | | Adjusted | | Unadjusted | | Adjusted | |
|  | RR | 95% CI | RR | 95% CI | RR | 95% CI | RR | 95% CI |
| Nonfrail | 1.00 | Reference | 1.00 | Reference | 1.00 | Reference | 1.00 | Reference |
| Prefrail | 1.16 | 1.09–1.24 | 1.17 | 1.10–1.26 | 2.99 | 1.99–4.48 | 3.18 | 1.95–5.18 |
| Frail | 1.35 | 1.20–1.51 | 1.36 | 1.18–1.58 | 5.91 | 3.47–10.09 | 2.90 | 1.32–6.35 |
| Female | 1.00 | Reference | 1.00 | Reference | 1.00 | Reference | 1.00 | Reference |
| Male | 0.92 | 0.87–0.97 | 0.97 | 0.89–1.05 | 1.71 | 1.26–2.32 | 1.09 | 0.64−1.86 |

RR: risk ratio; 95% CI: 95% confidence interval. For the estimation of the associations between influenza or the hospitalization and frailty, RRs and 95% CIs were adjusted for age, sex, marital status, educational attainment, equivalized income, household structure, smoking status, high-risk disease, influenza vaccination, civic participation, reciprocity, numbers of friends met in the last month, and the municipalities. For the estimation of the associations between influenza or the hospitalization and sex, RRs and 95% CIs were adjusted for frailty, age, marital status, educational attainment, equivalized income, household structure, smoking status, high-risk disease, influenza vaccination, civic participation, reciprocity, numbers of friends met in the last month, and the municipalities.

Supplemental Table 3 Interactions for frailty and sex in influenza incidents in the complete data.

|  | Influenza  (n=43348) | | | | Hospitalization due to influenza (n=43178) | | | |
| --- | --- | --- | --- | --- | --- | --- | --- | --- |
|  | Unadjusted | | Adjusted | | Unadjusted | | Adjusted | |
|  | RR | 95% CI | RR | 95% CI | RR | 95% CI | RR | 95% CI |
| Nonfrail female | 1.00 | Reference | 1.00 | Reference | 1.00 | Reference | 1.00 | Reference |
| Prefrail male | 1.09 | 0.97−1.24 | 1.05 | 0.91−1.21 | 1.13 | 0.48−2.68 | 1.35 | 0.51−3.56 |
| Frail male | 1.17 | 0.93−1.48 | 1.27 | 0.96−1.69 | 0.87 | 0.28−2.71 | 2.37 | 0.47−11.84 |

RR: risk ratio; 95% CI: 95% confidence interval. RRs and 95% CIs of the interactions were adjusted for frailty, sex, age, marital status, educational attainment, equivalized income, household structure, smoking status, high-risk disease, influenza vaccination, civic participation, reciprocity, and numbers of friends met in the last month, and the municipalities.
